# River depths and widths dataset for the territory of Poland

**DOI:** 10.1038/s41597-026-07537-0

**Published:** 2026-06-03

**Authors:** Tomasz Berezowski

**Affiliations:** Gdańsk University of Technology, Faculty of Electronics, Telecommunications and Informatics, Gabriela Narutowicza 11/12, 80-222 Gdańsk, Poland

## Abstract

The accurate estimation of critical river parameters, specifically width and depth, via remote sensing and machine learning techniques requires high-quality, geographically dense reference datasets for reliable validation and training. This paper presents a new, validated, high-resolution dataset for the territory of Poland, derived from field geodesy cross-section surveys conducted across 246 rivers between 2011 and 2013. The final processed dataset contains 20,782 records, providing referential measurements of river width, depth, water level, and center point coordinates, all closely aligned with near bankfull hydrological conditions. River widths range from 0.34 m to 2,384 m, while depths range from 0.005 m to 17.62 m. The quality control excluded outlying records, incomplete data, corrupted elevations, and infrastructure features. Validation against publicly accessible datasets confirms the data’s integrity: river depths align below the EU-DTM surface, river widths show strong correlation with MERIT Hydro (R^2^ = 0.93, RMSE = 35.1 m), and center points exhibit high positional accuracy relative to the MPHP10K map (median distance 1.8 m). This publicly available resource is applicable for training deep learning models (e.g., for river morphology estimation) and for validating large-scale hydrological and remote sensing products.

## Background & Summary

The morphology of rivers, such as their depth and width, is a fundamental parameter in a number of hydrological, ecological, and engineering applications. Accurate and comprehensive data on river depth and width is essential for hydraulic modeling and designing infrastructure. Moreover, the continuous measurement of river widths allows us to monitor their widening or narrowing globally, which is mostly due to morphological dynamics, reservoir construction, and climate change^1^. Despite its importance, large-scale, high-resolution, and uniformly standardized databases of river channel geometry remain scarce. This paper introduces the development, structure, validation, and potential applications of a novel, publicly accessible database of river depths and widths. This resource synthesizes geometric measurements from the territory of Poland under various environmental contexts, offering a tool for researchers and practitioners.

Width and depth of rivers can be derived from bathymetry data. While the process of measuring water depth dates back over 3000 years to Ancient Egypt^2^, modern bathymetric measurements utilize advanced techniques including GNSS, sonar^3^, and lidar^4^. These measurements are fundamental, as the accurate characterization of river width and depth is indispensable for understanding the movement of water and sediment through the landscape, serving as a critical constraint for hydraulic and hydrologic modeling. Yet, the availability of these measurements in public databases is limited mostly because they are labor-intensive in terms of acquisition and processing. Currently, the most comprehensive resource of detailed bathymetric measurements for rivers is the USGS 3D Elevation Program^5^, which, however, covers only several rivers in the USA.

An interesting solution to the river bathymetric data scarcity is offered by passive remote sensing such as satellite and aerial imagery. This approach, commonly termed satellite-derived bathymetry (SDB), infers depth by exploiting the interaction between sunlight and the water body^6^. A number of studies exist where SDB was utilized for rivers. The satellite and drone imagery were used to estimate pixel-based bathymetry for the Sacramento River^7^. A similar approach, but with the use of shallow neural networks, was applied to the Colorado and Potomac Rivers using satellite imagery^8^. In case of deep learning applications of SDB, a number of applications exist for seas^9–11^. Whereas applications of deep learning to river SDB are currently limited. This is likely due to not enough data being available to train and validate deep learning models for rivers.

Notably, SDB can also be obtained without in situ depth measurements. This is possible when using ICESat-2 data, as demonstrated in the Mekong River case study^12^. This method, however, requires a river discharge estimate to resolve the channel depth. An alternative approach involves fitting a hypsometric curve to surface water area and water level time series^13^. However, this technique cannot derive bathymetry below the water level corresponding to the minimum flow.

While river depth is derived from bathymetric data, river width often can be estimated using dedicated methods that use remote sensing data. River width estimates are essential inputs for estimating such attributes of fluvial systems as changes in river surface area, channel storage, and river discharge^14^. The initial step in river width estimation involves generating a precise water mask, typically accomplished using spectral indices. Once the river mask has been delineated, the next step is deriving a centerline. Finally, transects are calculated perpendicular to the centerline for width measurement^14–16^. The accuracy of this method is limited by the ground sampling distance of the satellite sensor used. While such an approach does not require river width data for calculation, a ground truth river width dataset is required for validation.

Currently, several resources exist with similar attributes. The Simple Global River Bankfull Width and Depth Database^17^, which was developed to provide global estimates of bankfull width and depth, along with confidence intervals. This product was derived using hydraulic geometry equations applied to the HydroSHEDS^18^ hydrography dataset. Another dataset is the Global River Widths from Landsat (GRWL), which utilizes Landsat imagery to provide global river width data^19^. The GRWL database generally covers rivers exceeding 30 m in width; however, the best accuracy was achieved for rivers wider than 90 m. A similar dataset is MERIT Hydro^20^, which has a global coverage and was developed to characterize the geometry of large rivers. This database provides attributes including flow direction, adjusted elevation, drainage area, river width, and height above the nearest drainage with a spatial resolution of ~90 m (3 arc-second). Yet another resource is the Surface Water and Ocean Topography (SWOT) River Database (SWORD), which was developed to serve as input data for SWOT products^21^. The SWORD database provides a global survey of rivers with such attributes as elevation, width, slope, and number of channels. An important advantage of the aforementioned resources is their global coverage, which enables applicability to large-scale hydrological modelling. However, each resource has some limitations. The GRWL and MERIT Hydro databases provide widths only for wider rivers, where water mask estimation was possible from satellite imagery. A similar limitation holds for SWORD, which was developed based on these two datasets. Conversely, for the Simple Global River Bankfull Width and Depth Database, validation showed errors up to 31% when compared to in situ depth measurements and up to 62% when compared to width derived from satellite imagery.

Given the need for data required to validate bathymetric and river width models and the limitations of currently available resources, a new dataset is proposed for the territory of Poland. The dataset was developed based on field surveys of river cross-sections, which provided detailed information about river centers, depths (0.005 m to 17.62 m), and widths (0.34 m to 2,384 m), as well as the water level at the time of measurement. The main applications of this dataset are the validation of river widths and depths for products characterized by less accurate estimates and for machine learning models’ training and validation.

The width, depth, water level, and location attributes were not estimated, but derived directly from the analysis of the river cross-sections. The cross-sections’ source is the field survey conducted in the period 2011-2013 using geodesy equipment. Because of that, the attributes of the dataset can be considered as referential. To support this, validation of the dataset was conducted using other publicly accessible datasets. The validation revealed that river depths align below the surface of the Digital Terrain Model, river widths are in close agreement with a state-of-the-art dataset estimated based on satellite imagery, and the center points of the measurements are located close to the reference river centerlines.

To ensure the quality of the dataset, outlying records were removed from the dataset. Moreover, records with missing data that was impossible to fill and with corrupted elevation were also removed from the dataset. Finally, for the sake of the consistency of the dataset, the records located directly at river infrastructure, such as bridges and dams, were removed from the dataset. Note that the records located close to the infrastructure were preserved in the dataset.

According to the Köppen classification^22^, the climate across most of Poland is humid continental (Dfb). In the westernmost regions along the Odra River and the Baltic Sea coast, the climate transitions to temperate oceanic (Cfb). Hydrologically, Poland is divided into seven distinct regimes^23^; for most of the territory, peak flows occur in March and April, while low flows occur in summer or autumn. An exception is found in high-mountain catchments, where low flows typically occur in winter.

River morphology in Poland is predominantly meandering or straight, though several rivers exhibit anabranched or braided characteristics^24^. While the majority of Polish rivers remain unregulated, distinct regulated reaches exist in the southern Odra catchment, the central and southern Vistula catchment, and the Vistula outlet^25^. Most of the country is drained by two major rivers, the Vistula and the Odra, with mean annual discharges (1950-2023) at their outlets of 1026 m^3^/s and 505 m^3^/s, respectively^26^. The suspended sediment load for the lower sections of both rivers is approximately 0.35 million tons/year^27^.

## Methods

### Source data

**Fig. 1:**
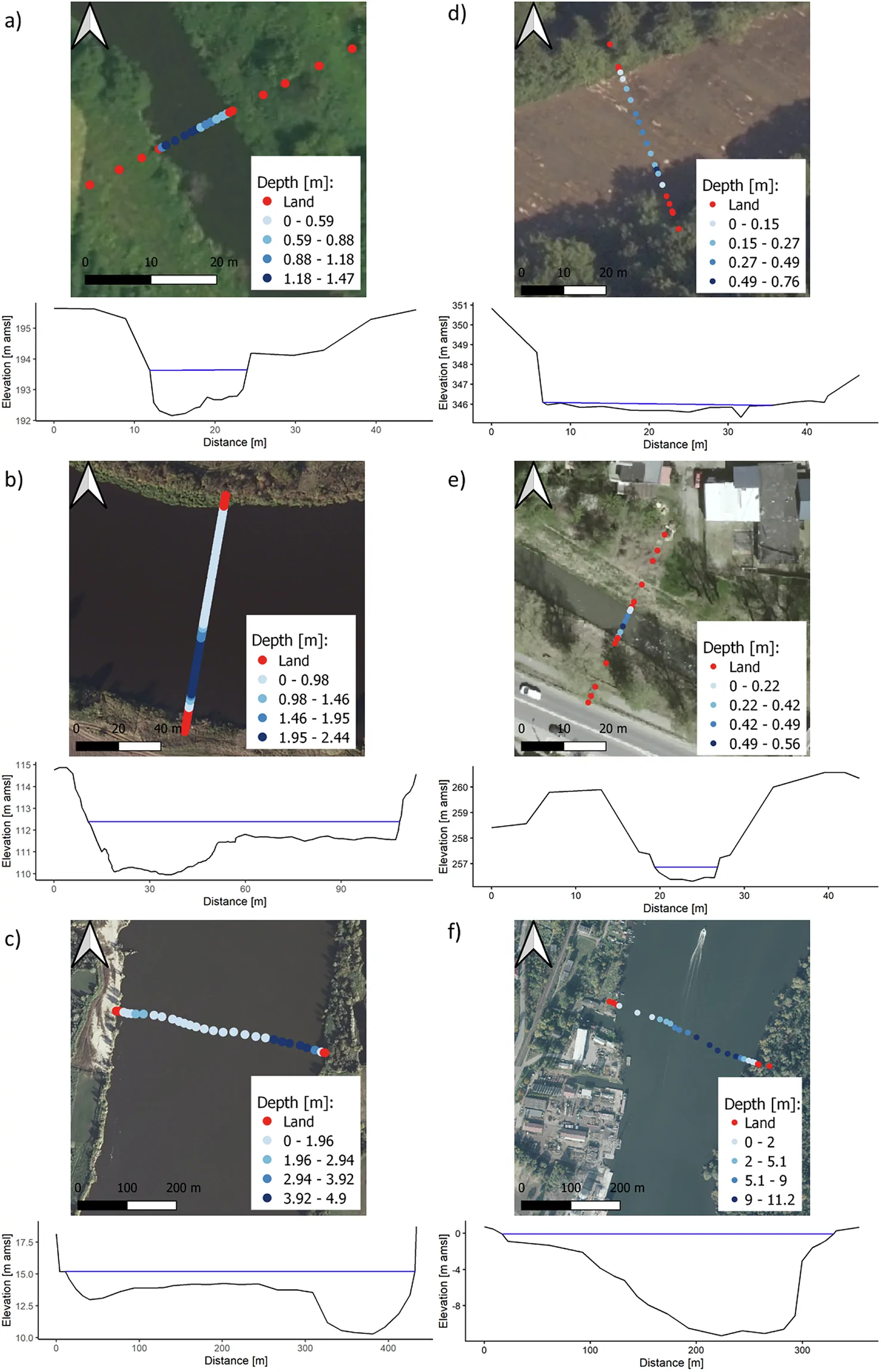
Examples of cross-section used in this study: (**a**) small river (upper Tanew, 22.952^°^E / 50.400^°^N, background image from 2012), (**b**) medium river (central Bug, 22.663^°^E / 52.385^°^N, background image from 2010), (**c**) large river (lower Vistula, 18.758^°^E / 53.537^°^N, background image from 2010), (**d**) natural mountain river (lower Oslawa, 22.202^°^E / 49.462^°^N, background image from 2015), (**e**) urban landscape river (central Brynica, 19.065^°^E/50.318^°^N, background image from 2012), (**f**) deep lowland river (lower Odra, 14.616^°^E / 53.485^°^N, background image from 2012).

The source data consisted of field survey cross-sections obtained through geodetic procedures for the purpose of hydrodynamic flood-risk modeling. The field survey used real-time kinematic global navigation satellite system (RTK-GNSS) devices, without any accompanying auxiliary measurements. Under ideal conditions, such as open areas, the accuracy of GNSS-RTK is 1-2 cm horizontally and 3-4 cm vertically at a 95% confidence level^28^. The river cross-sections were measured perpendicular to the centerline, using a sufficient number of points to accurately represent the riverbed geometry; simplified trapezoidal or triangular approximations were not permitted. In addition to the riverbed measurements, several land surface points were recorded extending up to 20 m from the riverbanks. The data covered 247 major rivers, channels, and their tributaries. In total, 23,286 cross-sections were measured using 931,557 points, yielding, on average, 40 points per cross-section. The distance between subsequent cross-sections was approximately 400 m. The representative examples of the cross-sections are presented in Fig. 1. These include three rivers of varying widths (small, medium, and large), a deep lowland river, and a comparison between a natural mountain river and a river from an urban landscape. To illustrate the variability of the original cross-section data used to generate the dataset, a representative sample of these sections is available in^29^. The field survey was conducted using the Polish coordinate reference system EPSG:2180 for horizontal coordinates and PL-KRON86-NH for vertical coordinates.

**Fig. 2:**
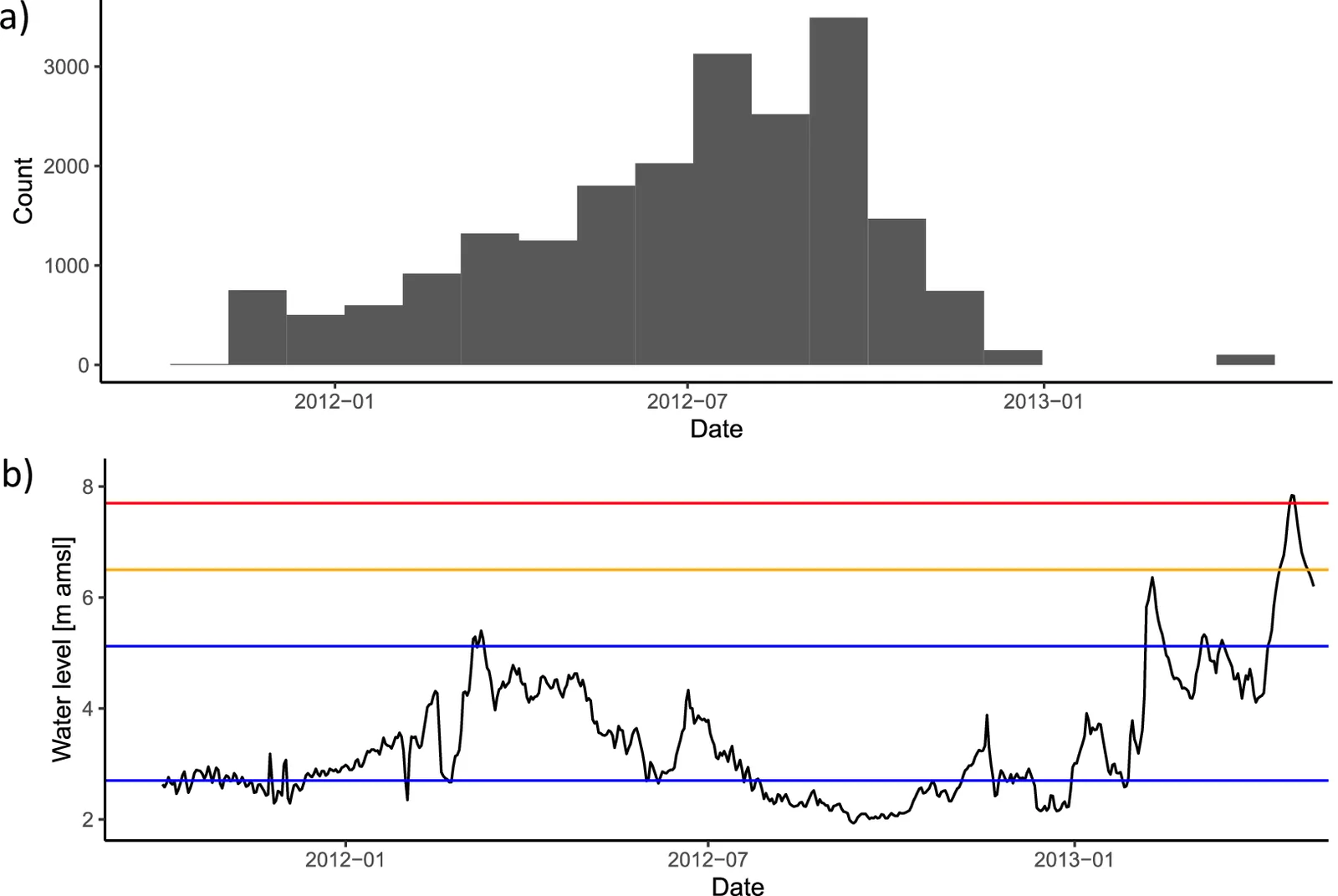
The distribution of the measurement dates in the cross-sections (**a**) and the water level in Vistula at Tczew (**b**), which is the profile closing the biggest catchment in the dataset. The water level plot is annotated by the lower and upper boundaries of the average water levels (blue), the warning water level (orange), and the alarm water level (red).

The field survey was conducted between October 2011 and April 2013. The majority of the measurements were conducted in summer and fall 2012. When analyzing the measurements in the scope of the water levels in the biggest catchments, they coincide with the period of average water levels (Fig. 2). Because of that, the water levels (and the derived depths) corresponded to near bankfull conditions. The exception was one river (Troja), measured in 2013 during high water levels. Yet, even for this river, the analysis of photographs taken during the field survey revealed that the water levels were slightly above bankfull.

The field survey measurements (x, y, and z coordinates) were accompanied by codes indicating whether the measurement was taken for a riverbed, surrounding terrain, or the water level. The water level codes were always taken directly at the intersections of the water surface and the banks. Another set of codes indicated the type of infrastructure covered by the measurements; these codes included e.g., dams, roads, fences, flood embankments, and others. Remaining attributes were: date of measurement, cross-section ID, photograph number, and river name.

The cross-sections data used to develop this dataset were provided by the National Council of Water Management (Krajowy Zarzad Gospodarki Wodnej - KZGW) in Poland, currently The State Water Holding - Polish Waters (Państwowe Gospodarstwo Wodne Wody Polskie). To contact the data provider (e.g. to obtain the primary data), please refer to the contact information at: https://www.gov.pl/web/wody-polskie/dane-kontaktowe. The author did not collect the primary data. On behalf of the National Council of Water Management, several subcontractors conducted the field measurements and primary data acquisition, including: “Okregowe przedsiebiorstwo geodezyjno kartograficzne Sp. z o.o. w Olsztynie”, “Centrum modelowania powodzi i suszy IMGW-PIB”, “Geokart-International Sp. z o.o.”, “HydroGIS Sp. z o.o.”, “Instytut morski w Gdańsku”, and “MGGP S.A.”. The dataset - comprising depths, widths, water levels, and their locations - is distributed with the consent of the State Water Holding - Polish Waters (ref. KPP.603.79.2025).

The source data were stored as EXCEL files (.xls) and, as the data were measured by several organizations, different field names, order, and formats were present in different files. For the most part, one file contained data for one river. However, for some cases, a few files contained data for the same river or, rarely, two rivers were in the same file. For some rivers, cross-sections covering entirely infrastructure (bridges, dams, etc.) were stored in a separate file, while for the remaining rivers, infrastructure and natural cross-sections were in the same file.

### Missing data

Even though the source data covered 247 rivers, some major Polish rivers were missing. This was the case for the San, Prosna, and Liwiec rivers. Another type of missing data was missing cross-sections for certain river reaches while data for upstream and/or downstream reaches were available. This was the case for the Odra River reach near Wrocław, the Warta River upstream of Poznań, Masurian and Warmia rivers near the Lake District, and a Vistula River reach upstream of its mouth. Since these data were missing entirely, there was no way to fill them; hence, the final dataset also lacks data for these rivers or river reaches.

The original data occasionally missed some attributes, most often river names and dates of measurement. In the case of these attributes, data were filled from preceding or following measurements, files containing cross-sections for infrastructure, or the photographs. If filling the missing data from other sources was not possible, the cross-section was removed from the final dataset.

### Corrupted elevation data

To highlight the corrupted elevation data, the z-coordinate of the water level was plotted against the European Digital Terrain Model (EU-DTM)^30^. This comparison clearly showed outlying measurements, which were then manually inspected. The analysis showed that the corrupted elevation was frequent in southern Poland, where the country boundary directly overlayed a river. For these cases, the elevation difference was up to 5 m in one or a few subsequent cross-sections; eventually, these cross-sections were removed from the dataset. A similar situation occurred, less frequently, for non-boundary rivers; these cross-sections were removed as well. The comparison with EU-DTM showed also that one river (Radew) had elevation data in an unknown vertical coordinate system, which had about 34 m difference from the expected PL-KRON86-NH system. Since the source coordinate system was unknown, the correction was impossible, and the entire river was removed from the final dataset.

### Data processing

The input files with cross-section data were distributed into groups containing the same headers. Next, each file was read into the R^31^ environment using the read_excel function from the readxl^32^ package. Following that, each cross-section was processed in the following way:

1. Codes of the measurements were analyzed to identify whether any type of infrastructure was present in the cross-section. The cross-sections containing such infrastructure were removed from the final dataset. The reason for this was that the purpose of the dataset was mainly to validate other products or to train machine learning models. Given this purpose, the end-user would expect that the cross-sections represent continuous river depths and widths, not locally outlying attributes due to the presence of infrastructure.
2. The number of water level markers (i.e. points indicating the water level at the river bank) was counted in each cross-section. Cross-sections with a different number of markers than two were removed from the final dataset because two markers were required to calculate a river width. This was the case where an odd number of markers was encountered, which represented invalid data. Another case was an even number of markers, but greater than two, which represented a cross-section with a narrow (smaller than 5 m) island. The cross-section with narrow islands was removed for consistency, because the original data contained separate cross-sections for each channel in a multi-channel river (if the islands were wider than 5 m).
3. The river width parameter was calculated as the Euclidean distance between the measurement points containing the water level codes.
4. The water level parameter was calculated as the mean z-coordinate from the measurement points containing the water level codes.
5. The river depth parameter was calculated by: (a) calculating the bottom elevation as the lowest z-coordinate between the measurement points containing the water level codes, and (b) subtracting the bottom elevation from the water level parameter (calculated in the previous step).
6. The center point coordinates of a cross-section were calculated by taking the median x- and y-coordinates of the measurement points in the cross-section. The median was chosen instead of the mean because the points were occasionally not uniformly distributed within a cross-section.

## Data Record

The dataset is available at Gdansk University of Technology - Bridge of Knowledge^33^ (https://doi.org/10.34808/n7xx-4171). The dataset is distributed as a single comma-separated value (.csv) file and its fields are described in Table 1.

**Table 1:** Description of the dataset fields.

Field name	Description
longitude	WGS-84 (EPSG:4326) longitude coordinate of the center point in decimal degrees
latitude	WGS-84 (EPSG:4326) latitude coordinate of the center point in decimal degrees
width	width of the river in meters
depth	depth of the river in meters
water_level	elevation of water level in meters above the mean sea level in PL-KRON86-NH
date	date of the measurements in the YYYY-MM-DD format for which the width, depth, and water level measurements are valid
name	name of the river in UTF-8 with spaces
id	unique identification number

## Data Overview

**Fig. 3:**
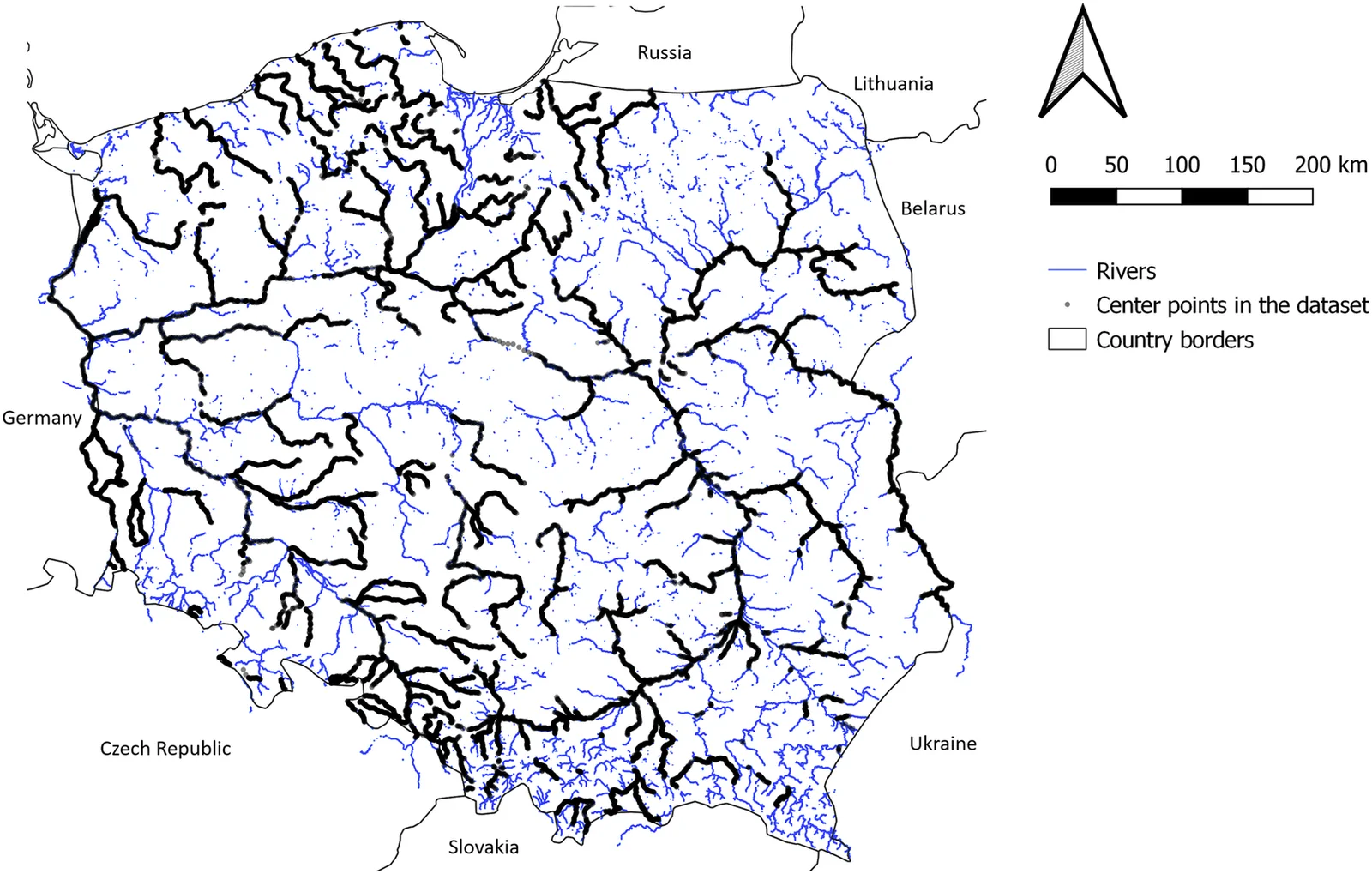
Distribution of the center points of the cross-sections available in the dataset across the territory of Poland.

In total, after the removal of missing, corrupted, or infrastructure data, the final dataset contained 20,782 records (Fig. 3), which was 11% less than the number available in the input files. The gaps induced by removal of the infrastructure points and other missing data were sparse. Because of that, their influence on hydrodynamic modelling is expected to be minimal. The exception is water damming infrastructure, which should be manually incorporated into the model setup.

**Fig. 4:**
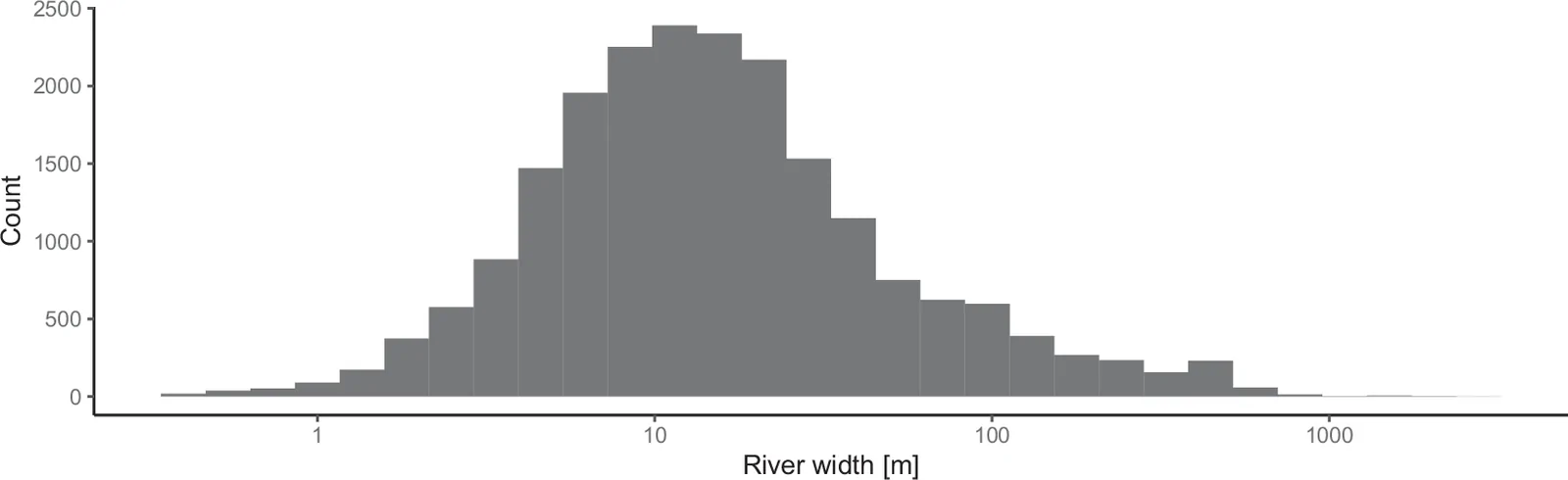
Distribution of the river widths available in the dataset. Note the x-axis in the log10 scale.

The river widths can be approximated by the log-normal distribution with the meanlog = 2.71 and sdlog = 1.20 parameters (Fig. 4). The mode of the widths is between 10 m and 11 m, and the central 50% of the data is between 6.8 m and 28.5 m. Rivers wider than 10 m (i.e., one Sentinel-2 pixel) account for 62% of the data, and rivers wider than 30 m (i.e., minimal width in the GRWL dataset) account for 24% of the data.

**Fig. 5:**
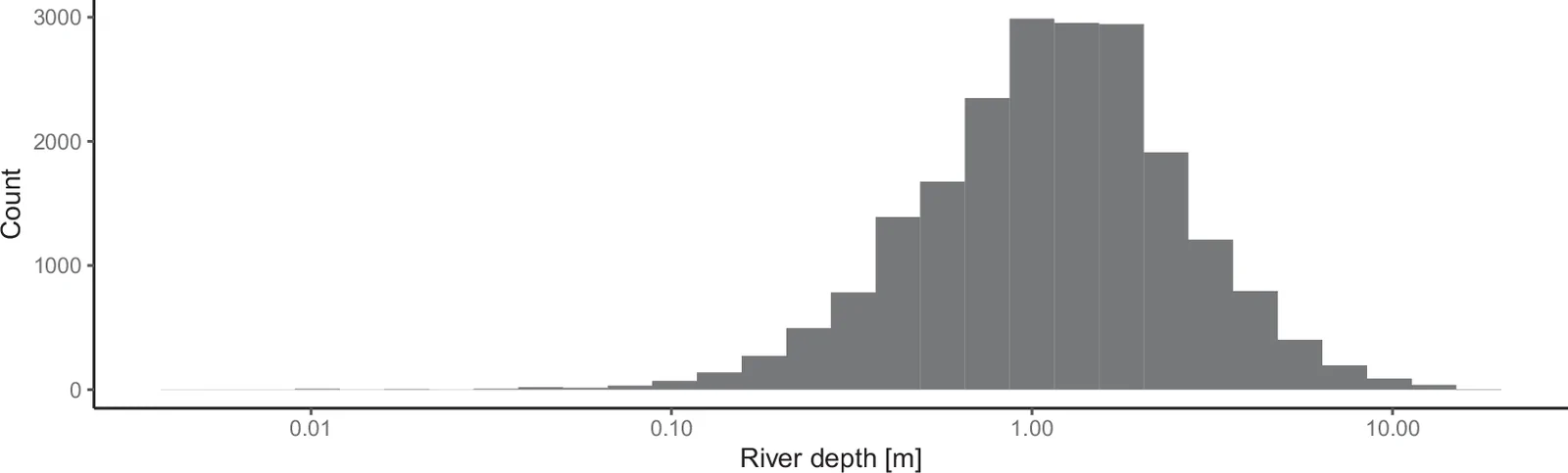
Distribution of the river depths available in the dataset. Note the x-axis in the log10 scale.

The river depths can be approximated by the log-normal distribution with the meanlog = 0.12 and sdlog = 0.83 parameters (Fig. 5). The mode of the depths is approximately 1 m, and the central 50% of the data is between 0.68 m and 1.93 m. The deepest rivers (above 10 m) represent artificially deepened profiles where rivers flow through ports located in the river mouth. The rivers in this dataset are in the depth range in which the remote sensing techniques of depth estimation are applicable, e.g.,^8^. However, due to turbidity or water color in some rivers, the applicability of these methods can be limited.

**Fig. 6:**
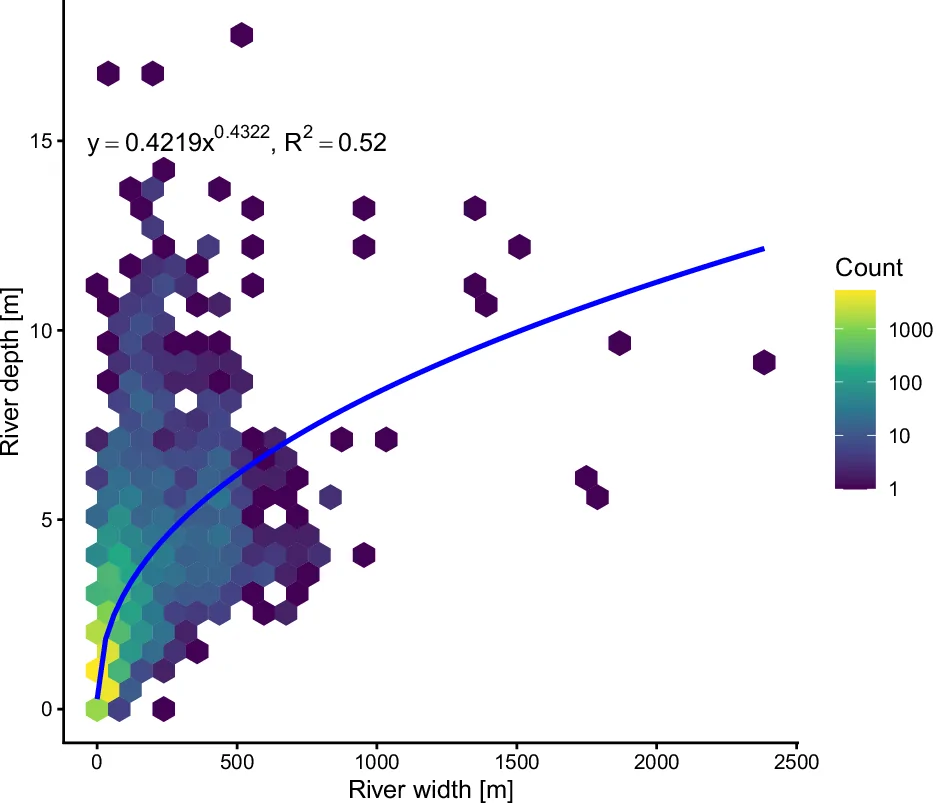
Relationship between river depths and widths in the dataset. The regression line is indicated in blue. Note that the hexagon fill color is in the log10 scale.

River depths are correlated with river widths in the dataset when the power-law relationship is analysed (R = 0.72, Fig. 6). Two clusters of river depth-width relationships can be identified. The first one, representing most data records, aligns with the regression line, and the second one, with a steeper relationship, represents narrower and deeper observations. The second cluster contains the deepest river reaches in the dataset, which include artificially deepened rivers that were present in port basins.

## Technical Validation

**Fig. 7:**
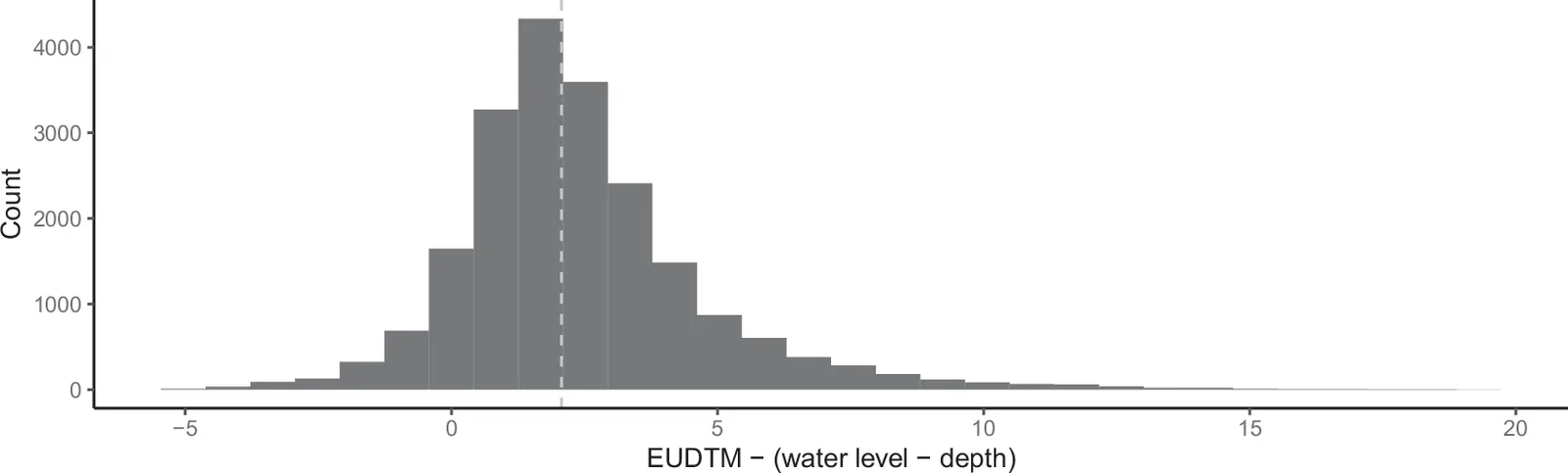
Distribution of river bottom elevation (river depth subtracted from water level) subtracted from the EU-DTM land surface elevation. The median is indicated by a dashed line.

The first validation task concerned river depths and water levels. In this task, it was validated whether the river bottom elevation (river depth subtracted from water level) would lie below the land surface elevation. As a reference land surface elevation, the EU-DTM was chosen. This was because this digital terrain model for the continental European Union was created using an Ensemble Machine Learning approach trained with about 9 million points from GEDI and ICESat-2 elevation data, overlaid against various existing digital surface models and land cover maps. Moreover, EU-DTM combined Random Forest, Cubist, and Generalized Linear Models to predict the most probable bare earth terrain height, which is characterized by an average error between +1 m and -2 m^30^. The validation revealed that the majority of river bottom elevations lay beneath the EU-DTM surface (Fig. 7). The median depth resulting from the subtraction of river bottom elevation from EU-DTM was 2.07 m and the mode was between 1 m and 2 m. Still, some river bottom elevations were higher than the EU-DTM surface. Manual investigation of these data records revealed no obvious pattern; hence, this difference is attributed to the error of EU-DTM, which can underestimate land surface elevations. It should be noted that this validation was indirect; specifically, it verified only whether the recorded depths were situated below the land surface elevation. A direct validation would require a comparison against an independent depth dataset. However, no such dataset was available for the study area at the time of this analysis.

**Fig. 8:**
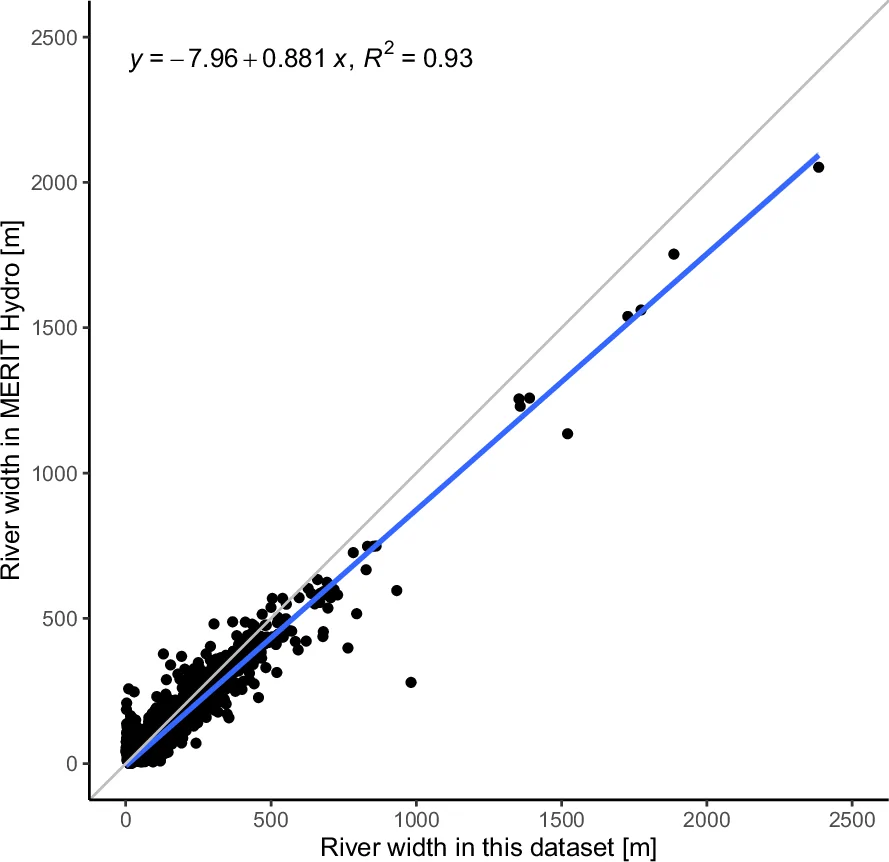
Relation between river widths in the MERIT Hydro dataset and widths in the dataset described in this paper (n = 5,773). The regression line is indicated in blue, and the 1:1 line is indicated in grey.

The second validation task concerned river widths. In this task, it was validated whether the river widths in this dataset matched river widths from a reference dataset. As a reference dataset, MERIT Hydro was chosen. This was because river widths in MERIT Hydro contains relatively narrow river widths in comparison to other publicly accessible datasets (e.g., GRWL, where the minimum river width is 30 m). MERIT Hydro is a raster dataset with a resolution of about 90 m. Since the river center lines derived in MERIT Hydro did not match exactly the center points in our dataset, only the center points that fell into a 90 m × 90 m pixel representing a river were taken into account in this analysis. The validation revealed that the relation between MERIT Hydro river widths and the river widths in this dataset is close to 1:1 (Fig. 8). The root mean square error (RMSE) and bias were 35.1 m and -16.4 m (MERIT Hydro was underestimating), respectively. The biggest difference between the datasets occurred at multi-channel rivers when one dataset identified one channel and the other dataset identified more than one channel.

River widths were also validated against the Polish hydrographic map at a scale of 1:10,000 (MPHP10K), selected for its temporal alignment with the current study. The MPHP10K includes the “rzeki_s” polygon GIS layer, representing the vectorized extents of major rivers. For validation, river widths were categorized into five equal-frequency classes. The territory of Poland was subdivided into 1^°^ × 1^°^ grid cells, and two transects per width class were measured within each cell using the “rzeki_s” layer. RMSE and bias were calculated based on these measurements (Table 2). The number of transects used for error calculation ranged from 92 to 99 per class, as not all width classes were present in every grid cell. While RMSE generally increased with river width, the 21-39.8 m class exhibited the lowest errors. This suggests that medium-sized rivers may have been vectorized with the highest precision in the MPHP10K dataset.

**Table 2:** RMSE and bias in river width classes.

River width range [m]	RMSE [m]	bias [m]	n
<8.8	0.96	0.26	92
(8.8–13.6]	1.43	0.21	94
(13.6–21]	2.32	0.13	99
(21–39.8]	0.77	0.00	95
>39.8	7.81	−0.28	94

**Fig. 9:**
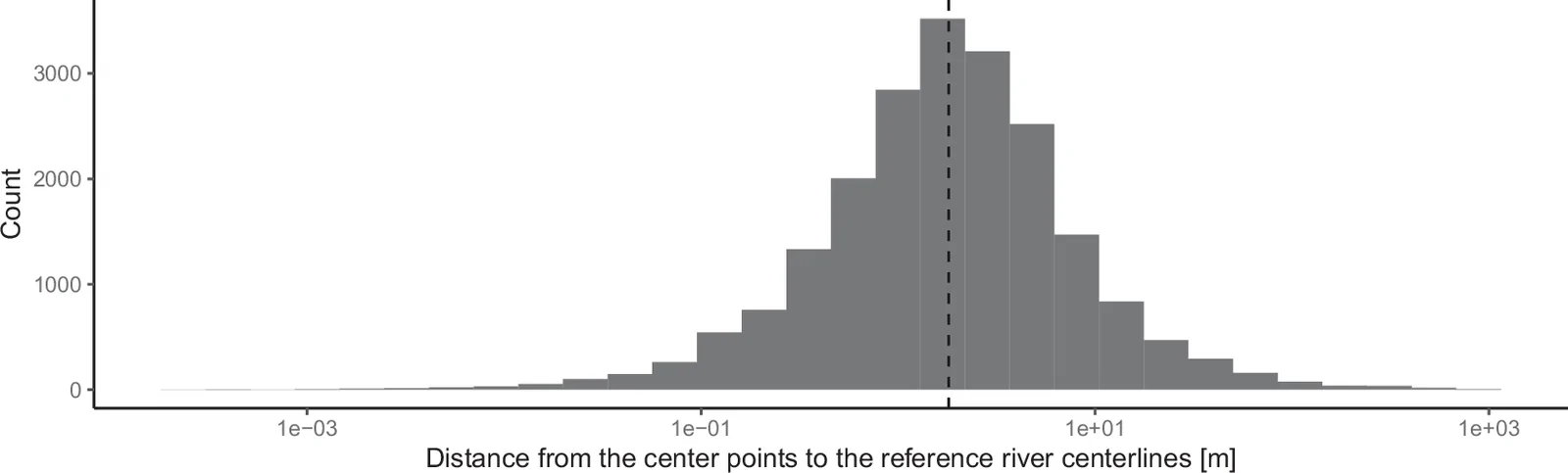
Distribution of distances between the river center points from this dataset and the river centerlines from the reference dataset. The median is indicated by a dashed line. Note the x-axis in the log10 scale.

The last validation task concerned river center points. In this task, it was validated whether the location of river center points in this dataset matched river centerlines from a reference dataset. As a reference dataset, again the MPHP10K was chosen. To perform this analysis, the “shortest line between features” tool in QGIS was chosen. The validation revealed that 95% of the distances are between 0 m and 18.4 m with the median (and mode) of 1.8 m (Fig. 9). The inspection of the outliers depicted that the remaining 5% of the distances occur in three cases: (1) most often, when the reference dataset had only one channel in a multi-channel river, (2) in ports, when cross-sections were measured in the port basins, distanced from a centerline, and (3) If a river was missing in the reference dataset.

As demonstrated in several European studies, river morphology - specifically width and depth - evolves over time^34^. Quantifying these changes across Poland remains challenging due to the absence of a comprehensive national-scale longitudinal study. However, a case study of the Biała River (southern Poland) indicates a width reduction of 16%–57% over a 131-year period^35^. Such temporal dynamics restrict the direct applicability of this dataset to studies aligned with the original measurement period (2011–2013). Specifically, the dataset is highly compatible with early Landsat 8 (launched 2013) and Sentinel-1 (launched 2014) observations. Conversely, morphological shifts may already be detectable in Sentinel-2 (launched 2015) data. Observations from the Surface Water and Ocean Topography (SWOT) mission, launched in 2022, may exhibit even more substantial deviations from the measurements presented herein due to the decade-long interval between surveys.

## Data Availability

The dataset described in this paper is publicly available at https://doi.org/10.34808/n7xx-4171.

## Code availability

The R functions used to generate the dataset are available in^36^ (https://doi.org/10.5281/zenodo.17609490).
